# From Isoprene Units to Polyprenols and Dolichols: 70 Years of Polyisoprenoid Biosynthesis Research

**DOI:** 10.1007/s12013-026-02112-1

**Published:** 2026-06-26

**Authors:** Zuzanna Bechler, Liliana Surmacz

**Affiliations:** 1 Institute of Biochemistry and Biophysics, Polish Academy of Sciences, Pawinskiego 5a, Warsaw, 02-106 Poland; 2 Doctoral School of Molecular Biology and Biological Chemistry, Pawinskiego 5a, Warsaw, 02-106 Poland

**Keywords:** Polyisoprenoid biosynthesis, Polyprenols, Dolichols, Prenyltransferases

## Abstract

Polyisoprenoids (polyprenols and dolichols) are a class of linear hydrophobic polymers present across all domains of life, serving essential functions for cellular viability and survival. Research on polyisoprenoids has progressed steadily since their discovery in the 1950s; however, these advances continue to highlight gaps in our understanding. This review provides a comprehensive overview of their biosynthesis, which occurs in three sequential stages: the formation of five-carbon precursors via the mevalonate (MVA) and methylerythritol phosphate (MEP) pathways, the elongation of the prenyl chain by prenyltransferases (PTs), and the final conversion into specific alcohol forms. Recent discoveries have substantially revised the classical understanding of dolichol biosynthesis, indicating a paradigm shift in the field. Furthermore, we discuss the essential biological roles of polyisoprenoids as lipid carriers for protein glycosylation, modulators of membrane fluidity, and key mediators in physiological defense systems against environmental stress, as well as their relevance to human disease and diagnostic applications. Finally, this article addresses critical knowledge gaps, focusing on the largely unexplored molecular mechanisms that regulate prenyltransferase activity and the yet-to-be-defined roles of these compounds in long-term stress adaptation.

## Introduction

**Fig. 1:**
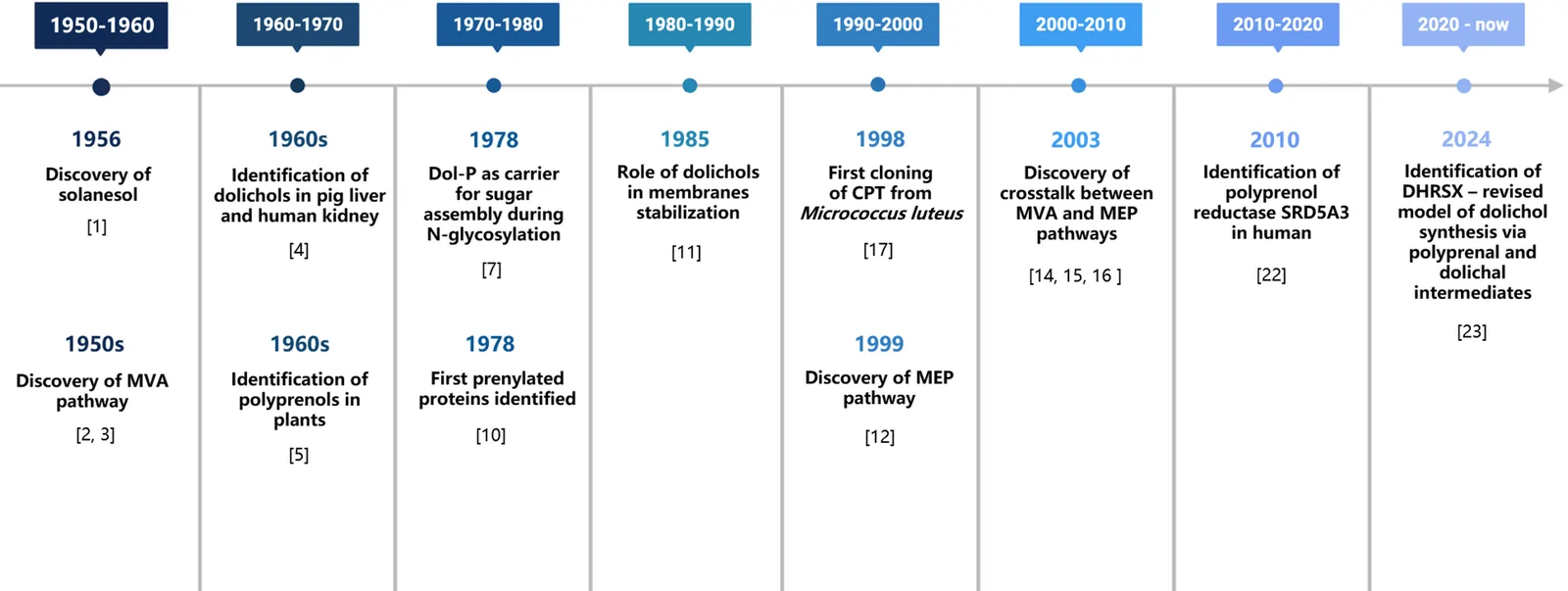
Key discoveries in polyisoprenoid biosynthesis over the past 70 years, highlighting major milestones in the field

Over the past seven decades, research on polyisoprenoids has evolved from a largely descriptive chemistry of isoprenoid lipids to the elucidation of complex biosynthetic pathways and essential cellular functions (Fig. 1). The history of this field began with the discovery of solanesol, the first plant polyprenol, in 1956 [1]. In parallel, intensive research led to the identification of the mevalonate (MVA) pathway as the primary route for the biosynthesis of isoprenoid precursors [2, 3]. Subsequent work expanded these findings to other plant polyprenols and animal dolichols, revealing the widespread occurrence of long-chain isoprenoid alcohols across all domains of life [4–6]. A major conceptual advance came with the discovery of dolichyl phosphate (Dol-P) as a lipid carrier of oligosaccharides in protein glycosylation, firmly establishing polyisoprenoids as indispensable components of cellular metabolism rather than minor secondary metabolites [7–9]. Further studies uncovered additional functions of polyisoprenoids, including their roles in protein prenylation [10] and as important components of cellular membranes contributing to membrane structure and stability [11]. The identification of the methylerythritol phosphate (MEP) pathway further expanded the landscape of isoprenoid biosynthesis, revealing alternative routes to isoprene precursors and their evolutionary diversification [12, 13], and highlighting metabolic crosstalk between the MVA and MEP pathways [14–16]. Progress in molecular biology led to the cloning of the first *cis*-prenyltransferases (CPTs) responsible for prenyl chain elongation [17–19], as well as the identification of accessory subunits such as the Nogo-B receptor (NgBR), which are essential for proper enzyme function and regulation in many eukaryotic cells [20, 21]. Recent advances have significantly reshaped our understanding of polyprenol-to-dolichol conversion. The identification of new enzymes and the proposal of a revised multistep model have challenged the classical view of the pathway. Together, these findings have led to a fundamental revision of dolichol biosynthesis in mammals and yeast, underscoring the complexity of this metabolic process [22–25]. This review summarizes the development of polyisoprenoid biosynthesis research, from isoprene units to final products.

## Polyisoprenoid Alcohols – Structure and Occurrence

**Fig. 2:**
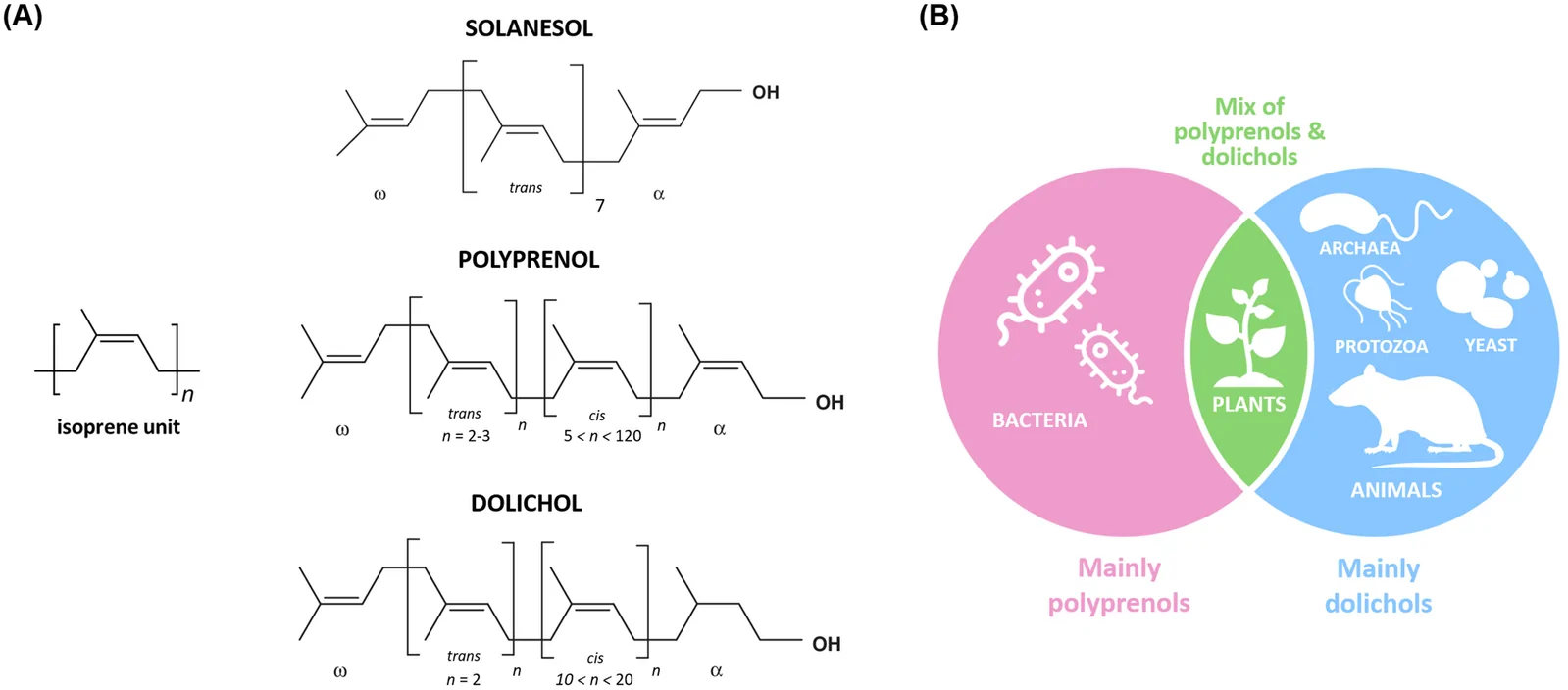
(**A**) Structural composition of a single isoprene unit, solanesol, polyprenol, and dolichol chains. *n* indicate the isoprene units in the *trans* and *cis* configurations, while α and ω denote the terminal isoprene units, respectively, (modified from [27]). (**B**) Distribution of polyprenols and dolichols across species, highlighting their characteristic occurrence patterns in different biological groups

Polyisoprenoids constitute a widespread class of linear hydrophobic polymers present in all organisms, ranging from prokaryotes (bacteria and archaea) to eukaryotes such as yeast, plants and animals. Those compounds belong to the vast isoprenoid family, which represents the most diverse group of natural compounds with more than 50 000 identified structures [26]. Structurally, polyisoprenoids consist of several to over 100 five-carbon (C5) isoprene units (i.u.) linked in a head-to-tail orientation. Their fundamental classification is based on the hydrogenation state of the α-terminal isoprene unit. Polyprenols (Pren) contain an unsaturated α-residue, whereas dolichols (Dol) are α-saturated compounds [27] (Fig. 2A). Beyond this distinction, structural diversity is further expanded by variations in chain length and the stereochemical configuration of internal double bonds. Based on the geometry of these bonds, polyisoprenoids are divided into three major groups: di*-trans-*poly*-cis*, tri*-trans-*poly*-cis* and all*-trans* [26]. Di*-trans-*poly*-cis* contains three internal *trans*-isoprene units, while the remaining internal units and the α-terminal isoprene unit are in the *cis* configuration, indicating formation via *cis*-addition to farnesyl diphosphate (FPP) – farnesyl type. Tri*-trans-*poly*-cis* contains four internal *trans*-isoprene units, while the remaining isoprene units are in *cis* configuration, indicating formation via *cis*-addition to geranylgeranyl diphosphate (GGPP) – a geranylgeranyl-type structure.

Polyisoprenoids are mainly accumulated as free alcohols and as esterified derivatives of carboxylic acids, whereas only a small fraction occurs in the form of phosphates [28]. Their occurrence, subcellular localization, and, importantly, the length of the synthesized polyprenyl chains are highly specific and vary considerably among different organisms, tissues, and cellular compartments.

Different nomenclature systems have been adopted in the literature for describing the chain length of polyprenols and dolichols. In the standard nomenclature, these compounds are designated, for example, as Pren-11 or Dol-11, where the numerical suffix indicates the number of five-carbon isoprene units present in the molecule. Alternatively, chain length may also be expressed as the total number of carbon atoms (e.g., C55) or as the number of isoprene units (e.g., 11 i.u.).

Polyisoprenoids most commonly occur in a *trans*/*cis* configuration. Polyprenols are primarily found in bacterial cells and plant photosynthetic tissues, whereas dolichols are characteristic of animal cells, yeast, archaea, protozoa and plant roots (Fig. 2B). These compounds may accumulate either as a single, well-defined molecular species or as homologous mixtures of polyprenols or/and dolichols with varying chain lengths, in which one homolog is usually dominant. The composition of these mixtures and the identity of the predominant homolog vary significantly between species and even among different tissues of the same organism.

Bacteria synthesize a single polyprenol, most commonly Pren-11, known as bactoprenol or undecaprenol [29–31]. However, a single dolichol species (Dol-12) has been identified in *Halobacterium halobium* [32].

Plants, particularly in their photosynthetic tissues, also accumulate polyprenols; however, their range is broad, spanning from 6 to over 130 i.u. In these organs, polyprenols occur as homologous mixtures forming either narrow families, typically consisting of 4–5 homologues, or broad distributions comprising more than 100 prenologue species [26, 33]. The most common leaf polyprenols in many plant species (e.g., Ficus sp., Magnolia sp., Euphorbia sp.) are medium-chain homologues ranging from 9 to 14 i.u., representing geranylgeranyl-type polyprenols [34]. In recent years, polyprenols with chain lengths of 9–11 i.u., with Pren-10 predominating, have been identified in chloroplast membranes of *Arabidopsis thaliana* and *Solanum lycopersicum*. These polyprenols influence membrane structure and fluidity, which are crucial for the proper functioning of photosynthesis [35, 36]. Long-chain polyprenols ranging from 14 to 25 i.u., have been detected in *Ginkgo biloba* [37], , *Taxus baccata*, *Abies koreana*, *Pinus strobus* and other plant species [34]. Plants of the genera Potentilla and Rosa typically contain two families of polyprenols: one in the range of 17–19 i.u. and another comprising significantly longer chains, extending up to approximately 50 i.u. The dominant homologues fall within the ranges of 23–29 and 30–35 i.u., respectively, depending on the genus [34]. The presence of even longer polyprenols, reaching up to 70 i.u., has been reported in members of the family Combretaceae (e.g., *Combretum molle*) [38].

Farnesyl-type polyprenols (betulaprenols), ranging from 6 to 9 i.u., have been identified in the woody tissue *of Betula verrucosa* [39]. Furthermore, a family of geranyl-type polyprenols (mono-*trans*, poly-*cis*), containing 10–12 i.u. and termed arachisprenols, has been identified in *Arachis hypogaea* [40]. In addition to typical polyprenols, latex-producing plants such as *Hevea brasiliensis*, *Taraxacum kok-saghyz*, *Parthenium argentatum*, and *Ficus elastica* accumulate extremely long polyisoprenes, typically comprising several thousand to over 10,000 i.u [41–45].

Dolichols are synthesized in all eukaryotic cells and accumulate as distinct dolichol families: one family in animals, two in yeast and up to three in plants, each consisting of a limited number of homologous species [35, 46–50]. Within each family, the number of homologs is relatively narrow, typically comprising about six to eight species [26]. Dolichol chain lengths generally range from 14 to 24 i.u., depending on the organism and tissue type. For example, Dol-19 dominates in humans [51], Dol-18 in rats [52], Dol-16 and Dol-21/23 in *Saccharomyces cerevisiae* and Dol-13, Dol-16 and Dol-21/23 *Arabidopsis thaliana* roots [46, 53]. Studies have demonstrated that the composition of long-chain dolichol families and the identity of the dominant homolog are not fixed but can vary depending on environmental conditions. In both *A. thaliana* hairy root cultures and *S. cerevisiae*, changes in growth conditions (sugar type and concentration or nutrient availability, respectively) induce a shift in the dominant dolichol between Dol-21 and Dol-23 [46, 53]. This suggests that dolichol chain-length distribution is dynamically adjusted in response to cellular metabolic status. These effects are thought to reflect alterations in the intracellular availability and balance of isoprenoid precursors, which influence flux through the polyisoprenoid biosynthetic pathway. In addition, growth-dependent metabolic conditions may affect the activity, processivity, and/or functional organization of enzymes responsible for polyisoprenoid chain elongation, thereby modulating the extent of polyisoprenoid chain elongation and resulting in changes in the relative abundance of individual dolichol species [46, 53].

A particular class of polyprenols includes all*-trans* compounds such as solanesol, found in *Nicotiana tabacum* [1] and members of the Solanaceae family [54], which contains 9 i.u., as well as spadicol, containing 10 i.u. and occurring in the family Araceae [28]. These compounds are notable in that they do not form homologous series but instead occur as discrete, individual molecules.

Hydrophobicity of prenyl chains determines their localization within cellular membranes. In mammalian cells, dolichols are found in all membranes, with the highest concentrations residing in lysosomes, Golgi apparatus, plasma membranes and endoplasmic reticulum [55, 56]. In plants, polyprenols accumulate within photosynthetic tissues in chloroplast thylakoids, whereas dolichols are mainly localized in the endoplasmic reticulum [26]. The accumulation of polyisoprenoids is a dynamic process influenced by developmental cues and environmental factors. Most notably, dolichol levels increase significantly during the lifespan in human brain, serving as a recognized biomarker for aging [57]. Also, in plants such as *Rosa chinensis* [58], *Kandelia obovate* [59], *Ginkgo biloba* and *Hevea brasiliensis* [42], the accumulation of polyprenols strongly depends on plant age. This age-dependent increase may be related to the limited understanding of enzymatic pathways involved in polyisoprenoid degradation or recycling, leading to their gradual accumulation during development. Consistent with this hypothesis, Weremczuk et al. [58] reported an increase in long-chain polyisoprenoids during leaf aging, suggesting that their accumulation may contribute to membrane stabilization and enhanced tolerance to oxidative stress under changing environmental conditions.

## Biosynthesis of Polyisoprenoids

Polyisoprenoid biosynthesis proceeds through three sequential stages. First, isopentenyl diphosphate (IPP) and dimethylallyl diphosphate (DMAPP) are synthesized as the universal isoprenoid precursors. Second, these intermediates undergo condensation rection, leading to elongation of the polyprenyl chain and formation of polyprenyl diphosphates (Pren-PPs). In the final stage, Pren-PPs are converted either into polyprenols or dolichols.

### Two Pathways of Isoprenoid Precursor Formation

**Fig. 3:**
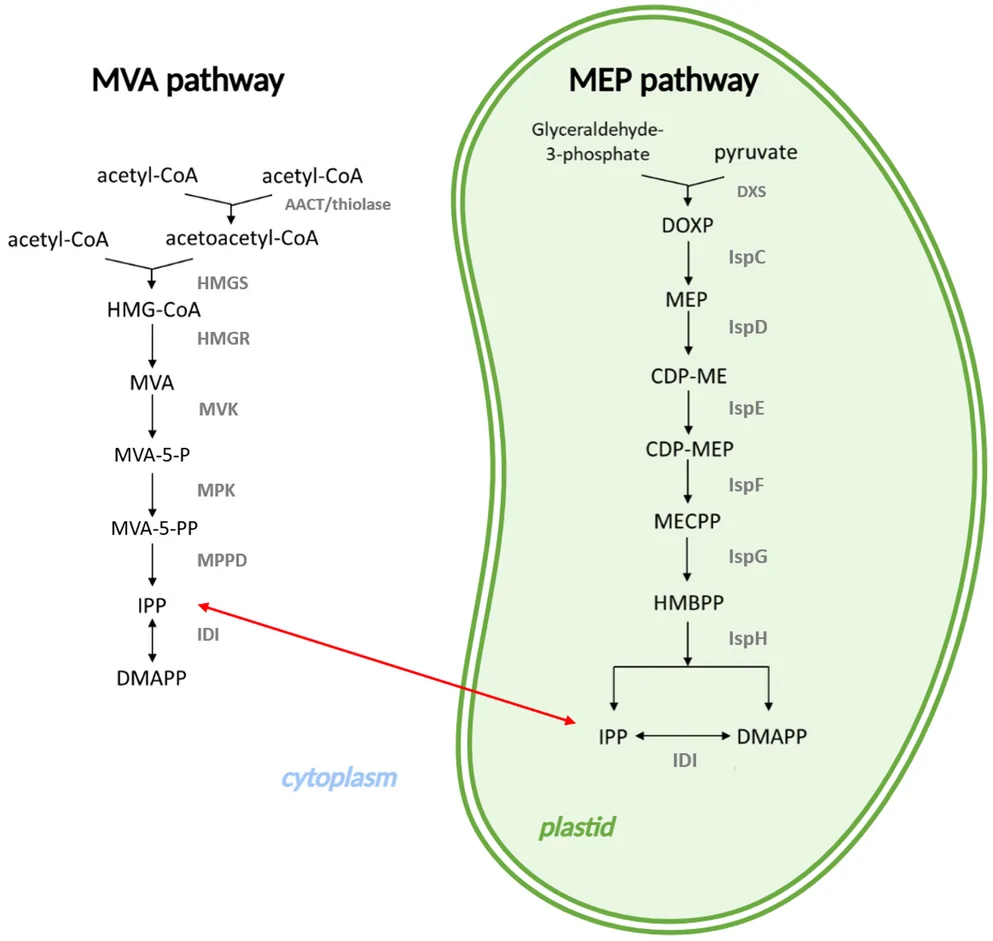
Biosynthesis of isopentenyl diphosphate (IPP) and dimethylallyl diphosphate (DMAPP) via the mevalonate (MVA) and methylerythritol phosphate (MEP) pathways, respectively, highlighting their distinct subcellular localization. The schematic illustrates two spatially separated metabolic routes responsible for the production of universal C5 isoprenoid precursors. The red double-headed arrow indicates intercompartmental exchange of IPP, reflecting metabolic cross-talk required to maintain precursor homeostasis for downstream terpenoid biosynthesis. The abbreviations for intermediates and enzymes used in the scheme are listed below. MVA pathway intermediates: HMG-CoA – 3-hydroxy-3-methylglutaryl-CoA; MVA – mevalonic acid; MVA-5-P – mevalonate-5-phosphate; MVA-5-PP – mevalonate-5-diphosphate. MVA pathway enzymes: AACT (thiolase) – acetyl-CoA acetyltransferase; HMGS – HMG-CoA synthase; HMGR – HMG-CoA reductase; MVK – mevalonate kinase; PMK – phosphomevalonate kinase; MPPD – mevalonate-5-diphosphate decarboxylase; IDI – isopentenyl diphosphate isomerase. MEP pathway intermediates: DOXP – 1-deoxy-D-xylulose 5-phosphate; MEP – 2-C-methyl-D-erythritol 4-phosphate; CDP-ME – 4-diphosphocytidyl-2-C-methyl-D-erythritol; CDP-MEP – 2-phospho-4-diphosphocytidyl-2-C-methyl-D-erythritol; MECPP – 2-C-methyl-D-erythritol 2,4-cyclodiphosphate; HMBPP – 4-hydroxy-3-methylbut-2-enyl diphosphate. MEP pathway enzymes: DXS – 1-deoxy-D-xylulose 5-phosphate synthase; IspC (DXR) – 1-deoxy-D-xylulose 5-phosphate reductoisomerase; IspD – 2-C-methyl-D-erythritol 4-phosphate cytidylyltransferase; IspE – 4-diphosphocytidyl-2-C-methyl-D-erythritol kinase; IspF – 2-C-methyl-D-erythritol 2,4-cyclodiphosphate synthase; IspG – HMBPP synthase; IspH – HMBPP reductase; IDI – isopentenyl diphosphate isomerase

The initial stage of biosynthesis is the formation of five-carbon building blocks of all polyisoprenoids: IPP and DMAPP. These precursors are synthesized from various compounds via two evolutionarily distinct and independent metabolic routes: the mevalonate (MVA) pathway and the methylerythritol phosphate (MEP) pathway (Fig. 3) [12, 60].

The MVA pathway takes place in cytosol and endoplasmic reticulum of eukaryotes (Fig. 3), including mammals and fungi, as well as archaea and certain Gram-positive bacteria [61]. It begins with condensation of two acetyl-CoA molecules, catalyzed by acetyl-CoA acetyltransferase (AACT/thiolase), to form acetoacetyl-CoA. A third acetyl-CoA is then added by HMG-CoA synthase (HMGS) to produce 3-hydroxy-3-methylglutaryl-CoA (HMG-CoA). This intermediate is reduced to mevalonate by HMG-CoA reductase (HMGR), which is the key regulatory enzyme of the pathway. Mevalonate is subsequently phosphorylated by mevalonate kinase to form mevalonate-5-phosphate, followed by a second phosphorylation step to generate mevalonate-5-diphosphate. This compound is then decarboxylated by mevalonate-5-pyrophosphate decarboxylase to generate IPP. In the final step, IPP is partially converted into DMAPP by IPP isomerase, which is required for DMAPP formation in this pathway [62].

The MEP pathway (Fig. 3) occurs in the plastids of plants and algae [63], as well as in most bacteria [64], cyanobacteria [65] and some Apicomplexa parasites [66]. It begins with the condensation of pyruvate and glyceraldehyde 3-phosphate to form 1-Deoxy-D-xylulose 5-phosphate (DOXP), catalyzed by DXS synthase. This product is then reduced to 2-C-methyl-D-erythritol 4-phosphate (MEP) by DOXP reductase (IspC). The pathway continues through a series of enzymatic steps involving multiple synthases and kinases. In the final stages, 2-C-methyl-D-erythritol 2,4-cyclodiphosphate (MECPP) is converted through reduction and elimination reactions catalyzed by 4-hydroxy-3-methylbut-2-en-1-yl diphosphate synthase (IspG) and 4-hydroxy-3-methylbut-2-enyl diphosphate reductase (IspH). These reactions produce both IPP and DMAPP directly. Therefore, unlike the MVA pathway, the MEP pathway does not require IPP isomerase for DMAPP synthesis. However, IPP isomerase may still function to balance the intracellular levels of IPP and DMAPP [62].

#### Metabolic Crosstalk between MVA and MEP Pathway

In plants, both isoprenoid biosynthetic pathways function in parallel; however, the discovery of “mosaic isoprenoids”, containing isoprene units derived from both the MVA and MEP pathways [13], provided strong evidence for metabolic crosstalk between these two routes and, consequently, their functional integration. Biochemical and molecular studies have demonstrated the exchange of precursors between cellular compartments, leading to the formation of such mixed-origin compounds [67, 68]. IPP is most frequently indicated as exchangeable intermediate, although other prenyl diphosphates, including FPP and GPP (geranyl diphosphate), and longer-chain oligoprenyl diphosphates, have also been suggested, particularly in the context of dolichol biosynthesis [14, 69].

The molecular mechanisms underlying this transport across the plastidial membrane remain poorly understood, and the involvement of specific membrane transporters has not yet been conclusively demonstrated [70, 71]. Metabolite exchange may occur in either a unidirectional or bidirectional manner and is regulated by developmental and light-dependent signals [72, 73]. Under certain conditions, such as during germination of *A. thaliana* prior to the full activation of the MEP pathway, or in root tissues where MEP activity is relatively low, a significant import of cytosolic precursors into plastids has been observed [73, 74]. However, this metabolic crosstalk is not sufficient to fully compensate for the loss of either pathway, as evidenced by the lethality of mutations in genes of the MVA or MEP pathways [67].

Stable isotope labeling experiments have confirmed that up to ~ 50% of isoprene units in dolichols may originate from the MEP pathway, despite their final biosynthesis occurring in the cytosol [60, 69, 75]. This metabolic plasticity, regulated by environmental signals, enables efficient redistribution of carbon flux and represents a key adaptive mechanism in plants. The interaction between the MVA and MEP pathways is characterized by a dynamic feedback system, in which changes in the activity of rate-limiting enzymes in one pathway can induce compensatory responses in the other. For instance, overexpression of HMGR in poplar leads to increased expression of MEP-related genes (DXS, DXR), whereas activation of the MEP pathway may result in downregulation of MVA-associated genes [60]. Under light conditions, the MEP pathway predominates, supporting the biosynthesis of carotenoids and chlorophylls, whereas in darkness the MVA pathway becomes more prominent, favoring phytosterols production [76].

In some bacteria, such as *Mycobacterium marinum*, the coexistence of both pathways serves as a backup system, enabling maintenance of isoprenoid levels under oxidative stress conditions [77].

In conclusion, the MVA and MEP pathways do not function as isolated parallel metabolic routes but rather as components of integrated regulatory network that provides homeostasis and flexibility in polyisoprenoid biosynthesis.

### Prenyl Chain Elongation via Prenyltransferases

MVA and/or MEP pathways generate IPP and DMAPP, which are utilized as substrates for polyisoprenoid biosynthesis through the stepwise elongation of polyprenyl chains. This process is catalyzed by a diverse family of enzymes known as prenyltransferases (PTs), which mediate the stepwise condensation of IPP onto an allylic diphosphate acceptor, generating linear hydrophobic polymers of defined length and stereochemistry [27].

Chain elongation is initiated by the condensation between DMAPP and IPP. The stereochemical configuration of each added isoprene unit - either *trans* (E) or *cis* (Z) - is determined by the specific PT involved. Accordingly, these enzymes are classified as *cis-* and *trans-* prenyltransferases (CPTs and TPTs), reflecting the spatial arrangement of isoprene units in their products [78].

The prenyl chain is then extended through successive condensation steps, resulting in polyprenyl diphosphates of defined chain length. These intermediates are subsequently converted into polyprenols or, in some cells, dolichols, which can be phosphorylated to their biologically active monophosphate forms (see section 'Conversion of Polyprenols and Dolichols to their Phosphate Forms'.). 

#### Chain Length Determination of Forming Polyisoprenoids

The final length of a polyisoprenoid chain is precisely regulated by the architecture of the PT’s active site. Structural studies and analyses of three-dimensional structures of PTs have led to the development of a model explaining polyprenyl chain-length determination. According to current structural models, the elongating polyprenyl chain enters a hydrophobic cleft within the enzyme during the second stage of biosynthesis. Chain elongation continues sequentially until the available volume within this internal pocket is entirely occupied. Then, the final product is released. Consequently, the specific size and geometry of this hydrophobic cleft serve as the determining factor for the final length of the resulting chain [79]. Beyond the enzyme’s internal architecture, several biochemical factors have been reported to modulate the product chain-length selectivity. Salt concentrations and divalent metal ion levels, as well as the relative ratio of allylic substrates to IPP co-substrates are determining factors [80]. Other membrane characteristics, including curvature, lipid composition, and fluidity, may also contribute to determining the product chain length [81].

#### Prenyltransferases – key players of polyisoprenoid biosynthesis

##### *trans*-prenyltransferases

*trans*-Prenyltransferases are systematically classified based on the carbon backbone length of the final synthesized product [78]. Most short-chain enzymes form homodimers, whereas medium- and long-chain forms can exhibit either homomeric or heteromeric subunit architectures [82].

**Fig. 4:**
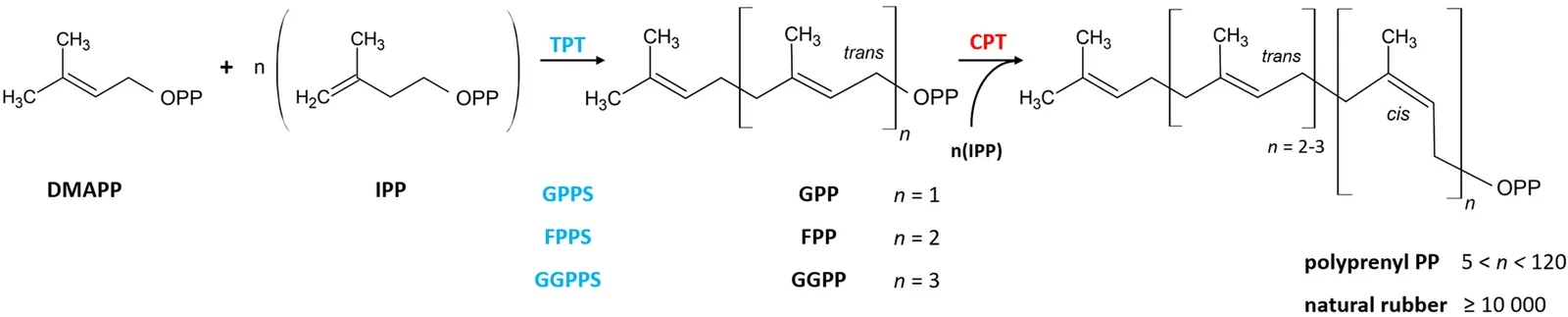
Schematic representation of prenyl-chain elongation initiated by DMAPP-IPP condensation and followed by successive IPP additions, catalyzed by *trans*- and *cis*-prenyltransferases (TPTs and CPTs), which determine both the stereochemistry (*trans* or *cis* configuration) of the double bonds and the chain length of the product. n denotes the number of isoprene units in the prenyl chain. Selected TPTs are shown: geranyl pyrophosphate synthase (GPPS), farnesyl pyrophosphate synthase (FPPS), and geranylgeranyl pyrophosphate synthase (GGPPS), which synthesize GPP, FPP, and GGPP, respectively. In contrast, CPTs generate long-chain *cis*-polyisoprenoids with variable n values, including polyprenyl diphosphate precursors and natural rubber chains (modified from [78])

The short-chain TPT family includes of geranyl (GPPS), farnesyl (FPPS), geranylgeranyl (GGPPS), and geranylfarnesyl diphosphate (GFPPS) synthases, which catalyze the formation of the corresponding prenyl diphosphates (Fig. 4). Precise control of isoprenoid metabolism is achieved through specific subcellular compartmentalization. GPPS is predominantly a plant-specific enzyme localized in plastids, where it catalyzes the condensation of DMAPP with one molecule of IPP to form GPP, a key precursor of monoterpenes, including essential oils. FPPS is widely conserved across all organisms and is localized mainly in the cytosol, while in plants it also functions as a central metabolic hub for generating cytoplasmic and mitochondrial isoprenoids [78]. FPPS catalyzes two consecutive condensation reactions: DMAPP and IPP first form GPP, which subsequently serves as an allylic acceptor for a second IPP condensation, yielding FPP [83]. FPP represents a central metabolic intermediate and a mandatory precursor for sterols, brassinosteroids, dolichols and ubiquinones. It also serves as a substrate for the biosynthesis of triterpenoids and sesquiterpenoids, as well as for the post-translational prenylation of specific proteins [78, 84]. GGPPS is present in both plants and animals and is mainly localized in the cytosol in animals, whereas in plants multiple isoforms occur in plastids and mitochondria. GGPPS catalyzes three sequential condensations of IPP with allylic substrates (DMAPP, GPP, FPP), resulting in the formation of GGPP. GGPP provides essential precursors for the biosynthesis of chlorophyll, carotenoids, and gibberellins, and also supports the formation of plastoquinones and phylloquinones, which function as key electron carriers in photosynthesis [76]. GFPPS is predominantly a plant-specific PT that catalyzes four sequential IPP additions to DMAPP, yielding geranylfarnesyl diphosphate (GFPP), a precursor of sesterterpenoids.

Medium-chain TPTs include hexaprenyl (HexPP) and heptaprenyl (HepPP) diphosphate synthases. These enzymes catalyze the addition of three or four isoprene units to FPP. They produce the precursors necessary for vitamin K2 biosynthesis. Structurally, these medium-chain enzymes are heteromeric complexes. Their enzymatic function depends on the physical association of two distinct subunits [85].

Long-chain TPTs are represented by solanesyl diphosphate synthases - SPS1 and SPS2. In the *A. thaliana*, these enzymes are localized in different compartments. SPS1 is found in the endoplasmic reticulum, while SPS2 is targeted to plastids. They synthesize solanesyl diphosphate for use in the hydrophobic side chains of ubiquinone and plastoquinone [86]. These prenylquinones are vital components for electron transport in both photosynthesis and mitochondrial respiration.

The first and second aspartate-rich motifs (FARM and SARM) are critical for catalysis. These motifs coordinate divalent magnesium ions to facilitate the binding of allylic substrates. The structural configuration and size of the catalytic pocket dictate the final prenyl chain length [82]. By restricting internal space, the active site functions as a molecular regulator or “molecular ruler”. This mechanism effectively terminates chain elongation once the appropriate product length is achieved.

##### *cis*-prenyltransferases

*cis*-Prenyltransferases constitute a conserved class of enzymes responsible for the biosynthesis of linear polyisoprenoid lipids across all domains of life. These enzymes utilize short-chain *trans*-prenyl diphosphates, primarily FPP or GGPP, as allylic primers and catalyze the sequential addition of IPP units in a *cis* (Z) configuration (Fig. 4). The direct products of CPTs are linear polyprenyl diphosphates (Pren-PPs), which exhibit characteristic mixed stereochemistry, forming di-*trans*-poly-*cis* or tri-*trans*-poly-*cis* structures depending on whether FPP or GGPP serves as the initial substrate [26]. Importantly, these products span a remarkably broad range of chain lengths, from short-chain (5–8 i.u), medium-chain (9–13 i.u), to long-chain species (≥ 16 i.u.), as well as extremely long polymers exceeding 10,000 i.u., such as those found in natural rubber [87–89].

In eukaryotic cells, CPTs are typically localized to the endoplasmic reticulum; however, some enzymes have been identified in lipid droplets as well as in plastids.

**Fig. 5:**
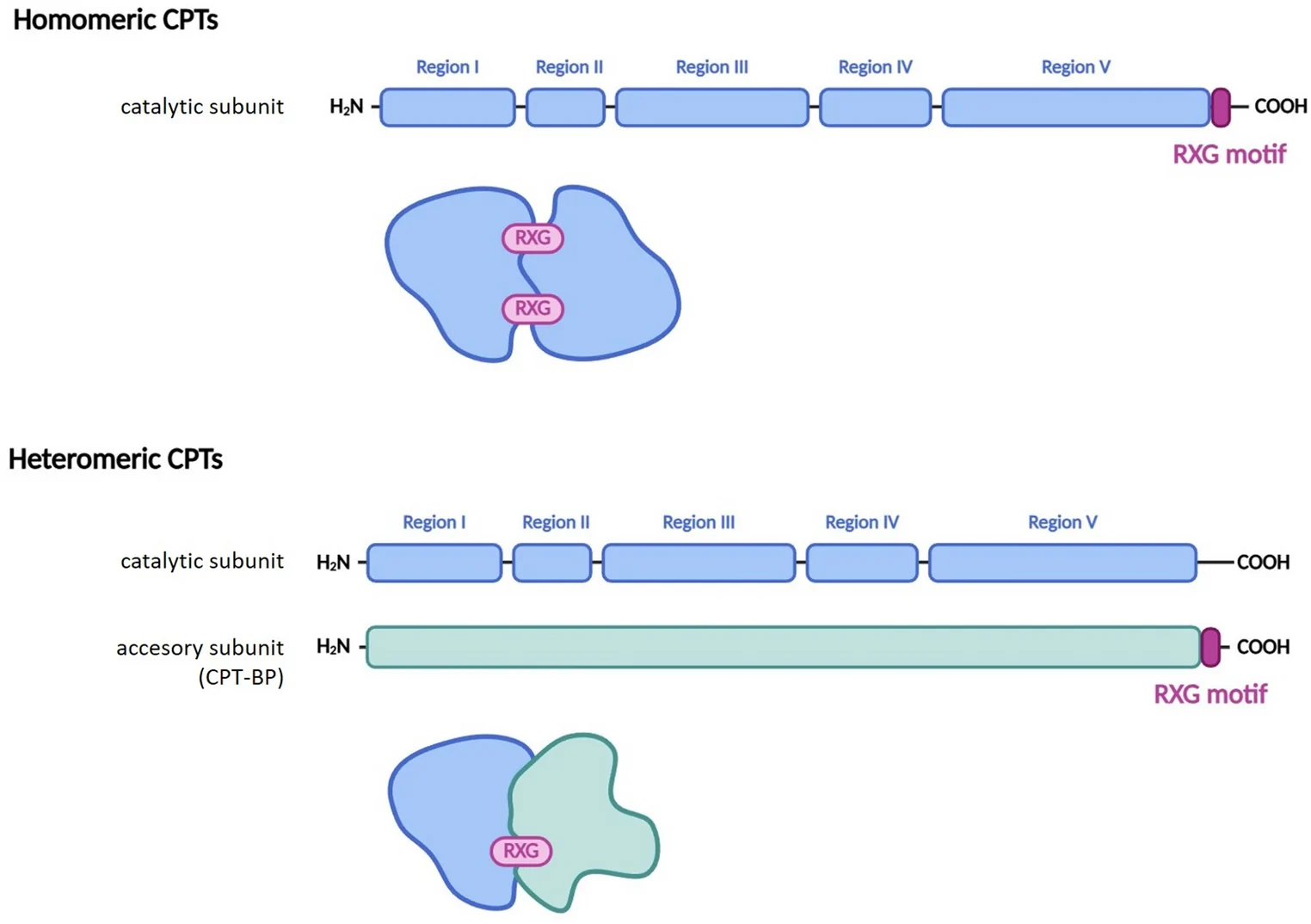
Classification of *cis*-prenyltransferases (CPT) into homomeric and heteromeric enzyme complexes, highlighting their structural organization and conserved sequence regions (Regions I–V), and the distribution of the RXG motif required for enzymatic activity. Homomeric CPTs form homodimers composed of identical catalytic subunits, each containing a C-terminal RXG motif that contributes to active-site integrity and enzymatic function. Heteromeric CPTs function as heterodimeric complexes consisting of a catalytic CPT subunit and a CPT-binding accessory protein. The catalytic subunit lacks the C-terminal RXG motif, which is provided by the accessory subunit and is essential for active-site formation and proper assembly of the functional enzyme complex

Based on their subunit organization, CPTs can be divided into homomeric and heteromeric complexes. Homomeric CPTs function as homodimers composed of two identical catalytic subunits and are capable of autonomous activity (Fig. 5). This group includes all characterized bacterial CPTs, as well as selected enzymes from plants, protozoa, and archaea. Many homomeric CPTs are involved in the synthesis of short-chain polyisoprenoid intermediates that serve as allylic primers for further elongation reactions. For example, the enzyme from *Mycobacterium tuberculosis* catalyzes the formation of (E, Z)-farnesyl diphosphate, which acts as a precursor for decaprenyl diphosphate synthesis [90]. Similarly, plastid-localized CPTs from *S. lycopersicum* (SlCPT1, -2 i -6) produce short-chain prenyl diphosphates (neryl-PP, nerylneryl-PP and *Z*,*Z*-farnezyl_PP), that function as intermediates in terpenoid biosynthesis [91].

The best-characterized homomeric CPTs are bacterial undecaprenyl diphosphate synthases (UPPS), identified in organisms such as *Escherichia coli*, where they synthesize bactoprenyl diphosphate (Pren-11) [92], an essential lipid carrier required for peptidoglycan assembly and protein glycosylation [93]. Homomeric CPTs are also present in plants, including enzymes from *S. lycopersicum* [36, 91] and *A. thaliana* [35, 48, 49], which produce polyisoprenoids of varying chain lengths, including short-chain (5–7 i.u.), medium-chain (9–13 i.u.), and long-chain species (> 18 i.u.), as well as in protozoa such as *Giardia lamblia*, which synthesizes products containing 11–12 i.u [94]. and in archaea such as *Methanosarcina mazei*, which produces prenyl chains shorter than 10 i.u [95].

In contrast, heteromeric CPTs act as heterodimers, in which catalytic subunit requires an additional protein partner, an accessory subunit, often referred to as a CPT-binding protein (CPT-BP, CBP), to assemble into an active catalytic complex (Fig. 5). Despite the presence of multiple CPTs within a single organism, typically only one CPT-BP can interact with different CPTs within that species. However, two CPT-BP homologs have been identified in the rubber-producing plants *Lactuca sativa* [96] and *Taraxacum kok-saghyz* [50].

Homomeric CPTs and CPT-BP contain a highly conserved RXG (or NXG) motif in their *C*-terminal region that is essential for enzymatic activity [89, 97]. In heteromeric CPT complexes, the catalytic subunit usually lacks this motif; therefore, interaction with a CPT-BP and formation of a heterodimer are required to provide the structural elements necessary for catalysis. The arginine residue within this conserved motif plays a key role by directly binding the IPP substrate [89]. Alterations within the *C-*terminal motif strongly reduce or abolish prenyltransferase activity in both homomeric and heteromeric systems [81].

In mammals and fungi, CPTs function exclusively as heteromeric complexes composed of a catalytic subunit and CPT-BP. In humans, the catalytic subunit DHDDS (called also hCIT, hCT) [98] forms an enzymatically active complex with NgBR [20, 99], whereas in yeast two distinct catalytic subunits, Rer2 and Srt1 form complexes with Nus1. These complexes exhibit distinct subcellular localizations that reflect their functional specialization. DHDDS-NgBR and Rer2-Nus1 are localized to the endoplasmic reticulum (ER), where their products act as lipid-linked sugar carriers essential for protein glycosylation. In contrast, Srt1-Nus1 is associated with lipid droplets, and its products are involved in the biosynthesis of the spore cell wall [100, 101]. Mutations in the DHDDS or NgBR genes lead to reduced levels of Dol-P and impaired protein N-glycosylation, which in humans manifests as congenital disorders of glycosylation type I (CDG-I) [20].

Formation of heteromeric CPT complexes with catalytic activity has also been demonstrated in numerous plant species, including *S. lycopersicum* (SlCPT3-SlCPTBP) [102], *A. thaliana* (AtCPT3-Lew1) [47, 103], *Panax ginseng* (PgCPT1-PgCPTL2) [104], and *L. sativa* (LsCPT1-LsCPTL2) [96]. Similar heteromeric systems have also been identified in protozoa, such as *Paramecium tetraurelia* (CPT1a-POC1) [105], and in archaea, including *Archaeoglobus fulgidus* (Af1219-Af0707) [106] and *Methanosarcina mazei* (MM_1083-MM_0618) [107]. Functional analyses indicate that the activity of these complexes is often essential for organismal viability. For example, the tomato SlCPT3–SlCPTBP complex and the *A. thaliana* AtCPT3-Lew1 complex are required for proper protein glycosylation, as their products serve as indispensable lipid-linked intermediates [47, 102, 108]. Polyisoprenoids synthesized by CPT-CPT-BP complexes are typically of medium chain length (approx. 15–19 i.u.) and function primarily as carriers in protein glycosylation pathways.

Heteromeric CPT systems have also been identified in plants producing natural rubber, such as *Hevea brasiliensis* and *Taraxacum brevicorniculatum*. In *H. brasiliensis*, HbHRT1 and HbHRT2 interact with HbHRBP [109], whereas in *T. brevicorniculatum*, TbCPT1, TbCPT2, and TbCPT3 form complexes with TbRTA [110]. In these species, in addition to CPTs involved in polyprenol/dolichol biosynthesis, specialized CPTs that participate in the production of natural rubber. However, CPT enzymes alone are not sufficient to synthesize extremely long polyisoprene chains. Instead, natural rubber biosynthesis is catalyzed by a specialized multimeric assembly known as the rubber transferase complex [111]. Additional proteins, including rubber elongation factor (REF) and small rubber particle protein (SRPP) play essential role in regulating polymer elongation. SRPPs serve as a structural scaffold that can recruit cytosolic CPTs to the endoplasmic reticulum (ER) or rubber particle membranes, facilitating the sequential condensation of IPP [112]. Beyond laticiferous tissues, homologs of REF/SRPP proteins, referred to as stress-related proteins (SRPs) or lipid droplet associated proteins (LDAPs), are widely distributed in non-rubber producing plants, including *A. thaliana* [113], suggesting broader roles in lipid metabolism and cellular stress responses. Overall, the CPT enzyme family exhibits a remarkable evolutionary diversification, with distinct members specialized for the synthesis of a wide range of polyisoprenoids. Despite extensive characterization across species, new variants and functions continue to be identified. However, the regulatory mechanisms governing CPT activity remain poorly understood and represent an important area for future research. Table 1 summarizes representative CPT enzymes from diverse organisms, highlighting their broad distribution and functional diversity.

**Table 1:** Summary of representative CPT enzymes from diverse organisms

*Homomeric CPTs*						
**Organism**		**Representative protein**	**Protein accession number (UniProtKB)**	**The chain length of synthesized products (number of i.u.)**	**Subcellular localization**	**References**
Bacteria	*Escherichia coli*	UPPS	P60472	11	cytoplasm	[29, 92]
	*Mycobacterium tuberculosis*	Rv1086	P9WFF5	10	cytoplasm ^+++^	[30]
	*Vibrio alginolyticus*	VacPT	MFH4454940 *	11	cytoplasm ^+++^	[54]
Archaea	*Methanosarcina mazei*	MM_0014	Q8Q0W6	9–10	cytoplasm ^+++^	[95]
Protista	*Giardia lamblia*	GlCPT	A8B1Z2	11–12	cytoplasm ^+++^	[94]
Plants	*Arabidopsis thaliana*	AtCPT1 (At2g23410)	O80458	18–23	ER	[35,48 , 49]
		AtCPT2 (At2g23400)	F4IMI8	ND	cytoplasm ^+++^	
		AtCPT6 (At5g58780)	Q8RX73	7	ER	
		AtCPT7 (At5g58770)	Q8GY03	9–11	plastid	
		AtCPT8 (At5g58782)	Q56Y11	ND	ER	
		AtCPT9 (At5g58784)	Q570Q8	ND	ER	
	*Solanum lycopersicum*	SlCPT1	C1K5M2	2	plastid	[36, 91]
		SlCPT2	K7WQ45	4	plastid	
		SlCPT4	K4D3U9	11	plastid	
		SlCPT5	K7 × 479	9–11	plastid	
		SlCPT6	K7W9N9	3	plastid	
		SlCPT7	K4CA50	7	plastid	
	*Lilium longiflorum*	LLA66	B2BA86	8–9	ER ^+++^	[198]
	*Rosa chinensis*	RcCPT2	A0A2P6QJX9	7–9	plastid	[58]
	*Lactuca sativa*	LsCPT2	A0A0A0QQH5	ND	ER	[96]
	*Taraxacum kok-saghyz*	TkCPT5	GWHPAAAA009126 **	5	plastid	[50]
		TkCPT6	GWHPAAAA009127 **	6–10	plastid	
		TkCPT7	GWHPAAAA026468 **	2	plastid	
		TkCPT8	GWHPAAAA020283 **	2	plastid ^+++^	

***CPT-Binding Proteins***						
**Organism**		**Representative protein**	**Protein accession number (UniProtKB)**	**Subcellular localization**		**References**
Archaea	*Archaeoglobus fulgidus*	Af0707	O29551	cytoplasm		[106]
	*Methanosarcina mazei*	MM_0618	Q8PZ76	cytoplasm		[107]
Yeast	*Saccharomyces cerevisiae*	Nus1	Q12063	ER		[21, 101]
Protista	*Paramecium tetraurelia*	POC1	A0CLN7	ER		[105]
Mammals	*Homo sapiens*	NgBR	Q96E22	ER		[20, 99]
Plants	*Arabidopsis thaliana*	Lew1	Q8H0V2	ER		[108]
	*Solanum lycopersicum*	SlCBP	A0A3Q7GXI0	ER		[102]
	*Rosa chinensis*	RcNus1	A0A2P6PP09	ER		[58]
	*Parthenium argentatum*	PaCBP	A0A384WDV7	ER		[199]
	*Lactuca sativa*	LsCPTL1	A0A0A0QLQ9	ER		[96]
		LsCPTL2	A0A0A0QP73	ER		
	*Taraxacum kok-saghyz*	TkCPTL1	GWHPAAAA045279 **	ER		[43, 50]
		TkCPTL2	GWHPAAAA041470 **	ER		
	*Hevea brasiliensis*	HbHRBP	P15252	ER		[109, 200]

***Heteromeric CPTs***						
**Organism**		**Representative protein**	**Protein accession number (UniProtKB)**	**The chain length of synthesized products (number of i.u.)**	**Subcellular localization**	**References**
Archaea	*Archaeoglobus fulgidus*	Af1219	O29049	8–12	cytoplasm	[106]
	*Methanosarcina mazei*	MM_1083	Q8PXY3	9–10	cytoplasm	[107]
Yeast	*Saccharomyces cerevisiae*	Rer2	P35196	15–17	ER	[21, 101]
		Srt1	Q03175	18–23	lipid droplets	
Protista	*Paramecium tetraurelia*	CPT1a	A0BQ08	16–18	ER	[105]
		CPT1b	A0D6J4	ND	ER	
Mammals	*Homo sapiens*	DHDDS	Q86SQ9	18–20	ER	[98]
Plants	*Arabidopsis thaliana*	AtCPT3 (At2g17570)	Q8S2T1	15–18	ER	[47, 108]
		AtCPT4 (At5g60510)	Q8LAR7	> 19	lipid droplets	
		AtCPT5 (At5g60500)	Q8LED0	ND	cytoplasm ^+++^	
	*Solanum lycopersicum*	SlCPT3	K7WCI9	15–18	ER	[102]
	*Rosa chinensis*	RcCPT1	A0A2P6PVA9	13–16	ER	[58]
		RcCPT3	A0A2P6RLA4	14–21	ER	
	*Parthenium argentatum*	PaCPT1	A0A384WD26	10–16	ER	[199]
		PaCPT2	A0A384WD08	10–16	ER	
		PaCPT3	A0A384WD98	10–16	ER	
	*Lactuca sativa*	LsCPT1	A0A0A0QM47	16–20	ER	[96]
		LsCPT3	A0A0A0QMT0	16–20	ER	
	*Taraxacum kok-saghyz*	TkCPT1	GWHPAAAA002594 **	> 20 000	ER	[43, 50]
		TkCPT2	GWHPAAAA002590 **	> 2 000	ER	
		TkCPT3	A0A3Q9VWW9	11–17	ER	
		TkCPT4	GWHPAAAA003168 **	11–17	ER	
	*Hevea brasiliensis*	HbHRT1	A5YTS0	> 2 000	ER	[109, 200]
		HbHRT2	Q8W3U4	> 2 000	ER	

For proteins not annotated in UniProt, accession numbers from databases containing the corresponding protein sequences are provided

* GenBank accession number; ** Genome Warehouse genomic accession number; ^+++^ in silico predicted subcellular localization; i.u. – isoprene units

### Formation of Polyprenols and Dolichols

**Fig. 6:**
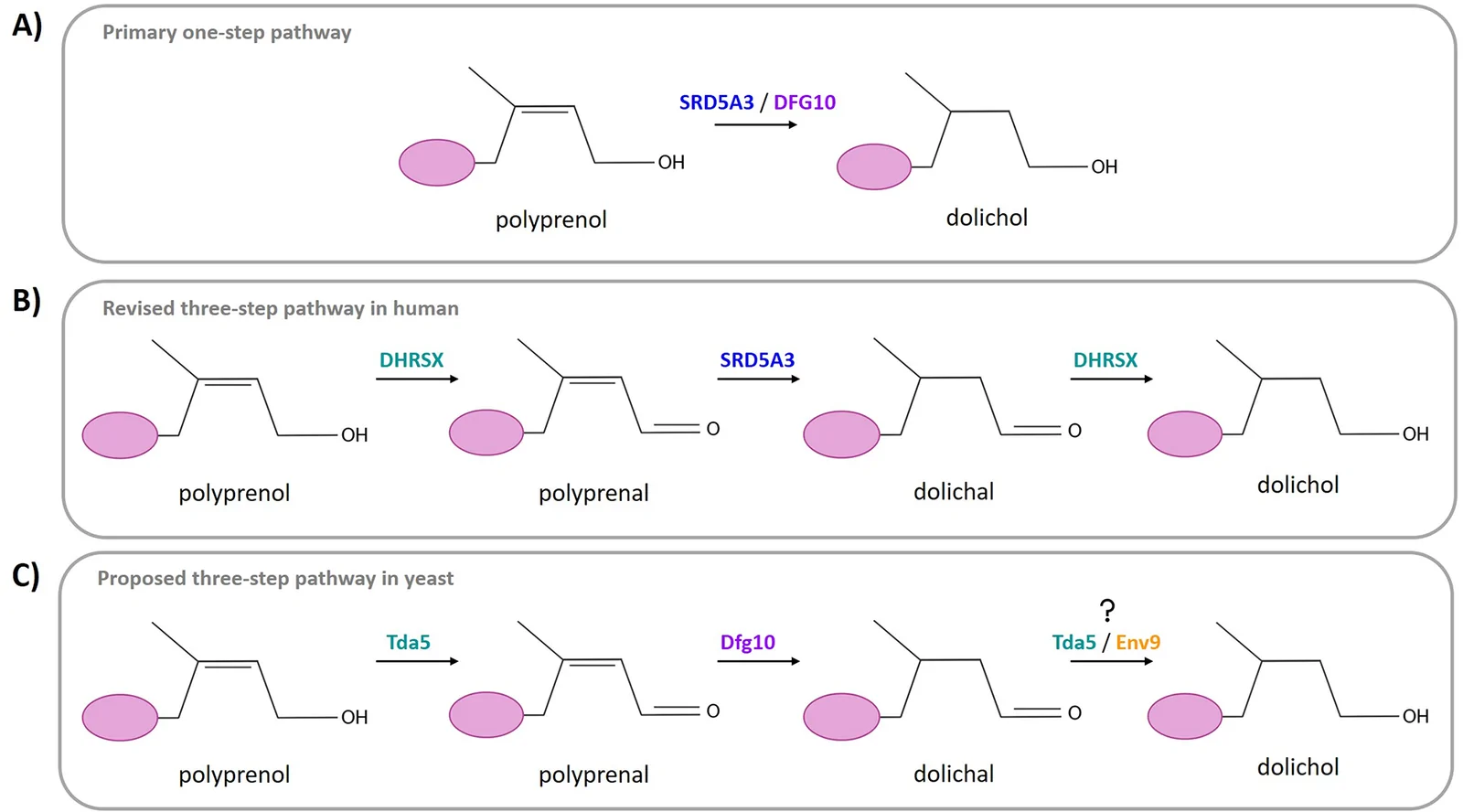
Comparison of the classical one-step and revised three-step pathways of dolichol biosynthesis in humans and yeast. (**A**) In the classical model, polyprenol is converted directly to dolichol in a single reduction step catalyzed by polyprenol reductase, SRD5A3 in humans and DFG10 in yeast. In contrast, (**B**-**C**) the revised pathway proceeds via a three-step mechanism involving polyprenal and dolichal intermediates. (**B**) In humans, this multistep conversion is mediated by dehydrogenase/reductase X-linked (DHRSX) [23], whereas (**C**) in yeast, two proteins, Tda5 and also Env9, are likely involved [23, 25]

In the final stage of polyisoprenoid biosynthesis, Pren-PPs, the end products of CPT activity, are converted into polyprenols and dolichols. Polyprenols are generated through dephosphorylation of Pren-PPs to free alcohols, in a reaction catalyzed by polyprenyl diphosphate phosphatase. Dolichols are generated from polyprenols via enzymatic reduction of the double bond within the α-isoprene unit, a modification that differentiates dolichols from their polyprenol precursors [36]. This terminal step in dolichol biosynthesis is catalyzed by polyprenol reductases. In humans, the reaction is mediated by polyprenol 5α-reductase type 3, encoded by the SRD5A3 gene, whereas in the yeast *S. cerevisiae* it is catalyzed by the Dfg10 protein [22]. Homologous proteins have also been identified in other species. *A. thaliana* possesses two closely related genes, PPRD1 and PPRD2. PPRD2 is essential for plant viability and its deficiency leads to plant sterility due to aberrant male gametophyte and sporophyte development [114]. However, this fundamental paradigm changed significantly in 2024, when it was shown that the conversion of polyprenol to dolichol in mammals is not a single-step reaction. Instead, it occurs through a more complex three-step pathway involving the enzyme DHRSX. Recent findings indicate that, in humans, dolichol biosynthesis proceeds via intermediate compounds, including polyprenal and dolichal, rather than through a direct conversion [23]. This research has fundamentally revised the classic model of dolichol biosynthesis by demonstrating that the enzyme SRD5A3 is unable to directly reduce the double bond of polyprenol alcohol, thereby supporting three-step pathway (Fig. 6). In the first step, DHRSX functions as a dehydrogenase, oxidizing polyprenol alcohol to an aldehyde intermediate known as polyprenal. Subsequently, SRD5A3 acts as a polyprenal reductase, utilizing NADPH to reduce the double bond and form dolichal. In the third and final step, DHRSX acts again, this time as a NADPH-dependent reductase, converting the terminal aldehyde group of dolichal back into an alcohol to produce dolichol [23]. The DHRSX-dependent pathway appears to be evolutionarily conserved across eukaryotes. Recent studies have identified the presence of a similar three step machinery in yeast. However, its enzymatic organization remains under debate. Some authors propose that Tda5 represents the yeast ortholog of human DHRSX [25], whereas others suggest that the dual role of human DHRSX is fulfilled by two distinct yeast enzymes: Tda5, which catalyzes the conversion of polyprenol to polyprenal, and Env9, which reduces dolichal to dolichol [24]. It remains unstudied whether plants utilize a comparable multi-step mechanism involving DHRSX-like intermediates, as observed in mammals, or whether they rely on alternative, more direct reduction pathways. Further research is required to elucidate this process.

The enzymatic machinery responsible for the conversion of polyprenol to dolichol is restricted to compounds synthesized in the cytoplasm and endoplasmic reticulum. In plants, polyprenols produced in plastids are not converted into dolichols, so no homologs of polyprenyl reductase are present in these organelles.

### Conversion of Polyprenols and Dolichols to their Phosphate Forms

**Fig. 7:**
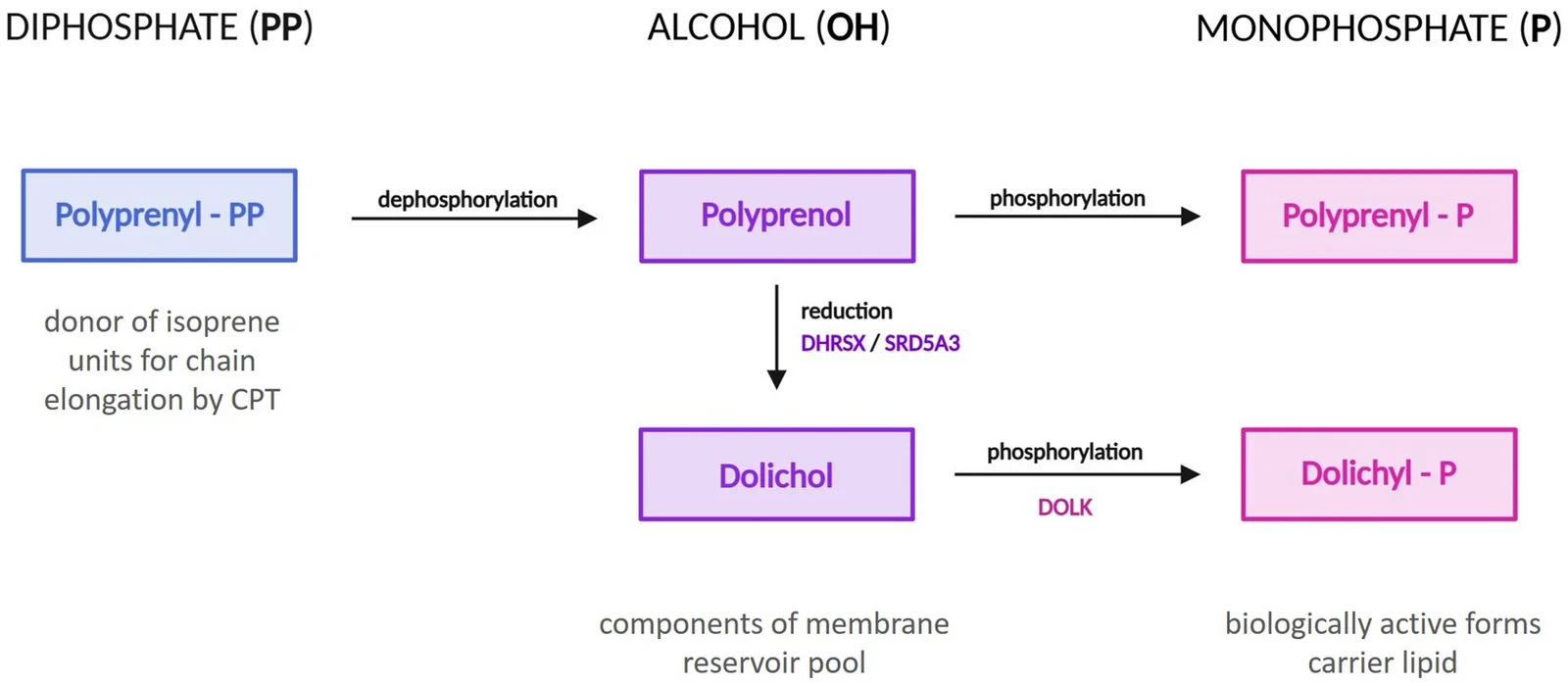
Schematic representation of the metabolic conversions of polyprenyl diphosphate into polyprenols, dolichols, and their monophosphate derivatives, highlighting distinct functional lipid pools and states. Polyprenyl diphosphate (polyprenyl-PP), synthesized by *cis*-prenyltransferase (CPT), represents the elongation product of polyisoprenoid chain formation and is dephosphorylated to polyprenol. Polyprenol can either be rephosphorylated to polyprenyl phosphate (polyprenyl-P) or reduced to dolichol. Dolichol is subsequently phosphorylated to dolichyl phosphate (dolichyl-P). Polyprenol and dolichol constitute the major cellular storage pool of polyisoprenoid lipids, whereas polyprenyl-P and dolichyl-P represent functionally active phosphorylated derivatives that serve as lipid carriers in glycan assembly. The enzymes shown correspond to the human pathway

Polyprenols and dolichols, formed as end products of polyisoprenoid biosynthesis, accumulate in cellular membranes as relatively inert alcohol forms. These compounds may act as lipid reservoirs and can be enzymatically phosphorylated to generate their biologically active derivatives, Pren-P (polyprenyl phosphate) and Dol-P. This conversion is necessary for their participation in cellular processes. Dol-P functions as an essential lipid carrier for glycan precursors during the early stages of protein N-glycosylation. The phosphorylation of free dolichol by dolichol kinase (DOLK) represents a key terminal step in the de novo biosynthesis of Dol-P (Fig. 7) [115].

In the early 1990s, studies in *S. cerevisiae* identified the *Sect. 59* gene as encoding dolichol kinase [116]. Earlier biochemical studies had detected CTP-dependent kinase activity in rat liver microsomes [117], bovine liver [118], and *Dictyostelium discoideum* [119]. The human DOLK was identified in 2002 [120], when a cDNA clone from a human brain library was identified as the mammalian homolog of yeast *Sect. 59*. In plants, dolichol kinase was first detected in soybean embryos [121].

The biochemical mechanism of DOLK in unique among kinases. While most kinases utilize ATP as a phosphate donor, DOLK uses cytidine triphosphate (CTP) [122]. The enzyme contains fifteen transmembrane domains [123] and a conserved DXXAXXXGXXXGX8KKTXEG motif that forms a CTP-binding site [122]. Structure-function studies indicate that this C-terminal domain is exposed to cytoplasm and is essential for catalytic activity. Within this motif, the specific glycine residues, G420 in yeast and G443 in humans, are essential for catalytic function [122]. Deficiencies in human DOLK result in a group of severe congenital disorders, as described in section 'Disruption of the Dolichol Biosynthetic Pathway and its Consequences in Humans' .

In plants, dolichol kinase plays a fundamental role in growth and reproduction. The *A. thaliana* gene AtDOK1 encodes a functional dolichol kinase capable of rescuing the defects of yeast *Sect. 59* mutants. Complete loss of DOK1 function in Arabidopsis is lethal, indicating that dolichol phosphorylation is essential for Dol-P production in this species [124, 125]. Heterozygous *dok1* mutants exhibit defects in male and female gametophyte development, leading to low pollen viability and shortened siliques [124]. Furthermore, partial knockdown of *AtDOK1* results in early flowering, suggesting that Dol-P availability influences the transition from vegetative to reproductive growth [125].

The conversion of dolichol into Dol-P represents a central step eukaryotic cell biology. By activating membrane-associated dolichol, DOLK ensures a significant supply of lipid carriers essential for essential glycosylation processes and overall cellular homeostasis.

## Biological Function of Polyprenols and Dolichols

Polyisoprenoids play essential roles in many basic cellular functions across all domains of life. Some of their roles are well established, while others still require further investigation. Polyisoprenoids are essential for cellular viability and survival. Polyisoprenoid phosphates function as indispensable hydrophobic lipid carriers and cofactors in protein glycosylation [62], and assembly of glycosylphosphatidylinositol (GPI) anchors [126]. Beyond these metabolic functions, polyisoprenoids modulate the properties of biological membranes by increasing their fluidity and permeability [27]. They also act as key mediators in physiological defense systems, including antioxidants and free-radical scavengers activity that protect cells from reactive oxygen species (ROS) and lipid peroxidation [127]. In plants, they are particularly important for environmental adaptation to changing conditions, helping to maintain proper functioning under stresses such as high temperature [128], salinity [129], and pathogen infection [130]. Disruption of dolichol biosynthesis in humans leads to severe congenital disorders, highlighting the critical importance of polyisoprenoid metabolism in human health and disease [131–133].

### Protein Glycosylation

**Fig. 8:**
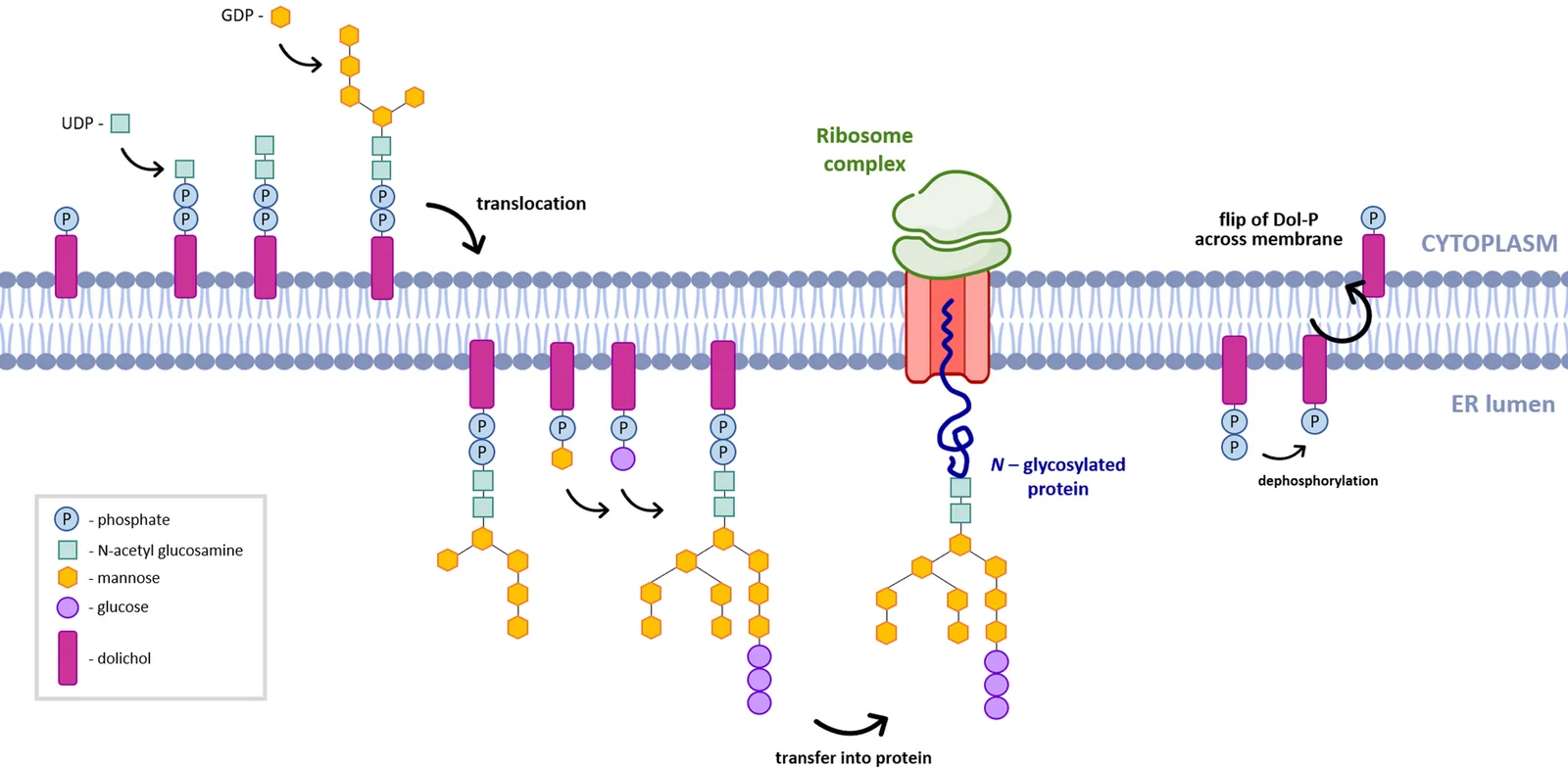
Schematic representation of the protein N-linked glycosylation pathway in the endoplasmic reticulum (ER). The illustration depicts the stepwise assembly and transfer of an oligosaccharide precursor on a dolichol phosphate (Dol-P) lipid carrier. The process initiates on the cytosolic face of the ER, where N-acetylglucosamine and mannose residues are sequentially added to Dol-P. The lipid-linked intermediate is then translocated into the ER lumen, where the glycan is further extended by the addition of mannose and glucose residues. The fully assembled oligosaccharide is transferred en bloc to a target protein. Following transfer, dolichol diphosphate (Dol-PP) is dephosphorylated to Dol-P and recycled back to the cytosolic leaflet of the ER membrane for subsequent glycosylation cycles

The role of phosphorylated forms of polyisoprenoids, Pren-P and Dol-P, play essential roles in protein glycosylation (Fig. 8) in prokaryotic and eukaryotic cells, respectively, where they act as membrane-bound lipid carriers that enable the stepwise assembly of oligosaccharide chains [26, 62, 89, 93, 134, 135].

Biosynthesis of lipid-linked oligosaccharides (LLOs) is initiated on Dol-P localized in the endoplasmic reticulum membrane. The first step involves the transfer of N-acetylglucosamine-1-phosphate (GlcNAc-1-P) from UDP-GlcNAc to Dol-P. In this process, the sugar is transferred together with its phosphate group, resulting in the formation of dolichyl diphosphate N-acetylglucosamine (Dol-PP-GlcNAc). Importantly, the second phosphate of the diphosphate linkage originates directly from the sugar donor, creating a diphosphate bond between dolichol and the glycan moiety rather than through a separate phosphorylation step. Subsequent elongation of the oligosaccharide proceeds through the sequential addition of monosaccharides from nucleotide-activated donors, such as UDP-GlcNAc and GDP-mannose, on the cytoplasmic side of the membrane. After partial assembly, the intermediate is translocated into the lumen of the endoplasmic reticulum, where further extension occurs using dolichyl phosphate-linked sugar donors, including Dol-P-Man and Dol-P-Glc. Throughout this process, the oligosaccharide remains linked to dolichyl diphosphate (Dol-PP), which serves as a stable lipid carrier until the completed glycan is transferred en bloc to an asparagine residue within the consensus sequence Asn-X-Ser/Thr by the oligosaccharyltransferase (OST) complex. The resulting glycoprotein undergoes further maturation in the ER and Golgi apparatus. After transfer of the oligosaccharide to the nascent protein, Dol-PP remaining in the ER membrane is dephosphorylated to Dol-P by membrane-associated phosphatases. Dol-P is subsequently recycled into the lipid-linked oligosaccharide biosynthesis pathway for further rounds of N-glycosylation (Fig. 8). The availability of Dol-P is a key factor controlling the efficiency of glycosylation [62].

Dol-P also participates in other types of glycosylation. In O-glycosylation, glycans are transferred to proteins and attached via O-glycosidic bonds formed between the glycan and hydroxyl groups of amino acid side chains, primarily serine and threonine. One specific form is O-mannosylation, in which Dol-P serves as a lipid carrier of mannose residues, similarly to its role in N-glycosylation [136]. Dol-P is also involved in C-mannosylation, where it donates a mannose residue that becomes covalently attached to the C2 position of the indole ring of tryptophan [137].

Protein glycosylation is essential for the proper functionality of glycoproteins, as it influences multiple physicochemical and functional properties, including protein folding, stability, intracellular trafficking, secretion, and subcellular localization [138]. Glycoproteins are involved in a wide range of biological processes, including molecular recognition, cell adhesion, cellular signaling, fertilization, immune responses, and host-pathogen interactions [139]. Furthermore, alterations in glycosylation patterns often lead to significant changes in protein activity and function. Impaired glycosylation pathways are critically linked to the progression of various pathological states, including autoimmune, oncological, and neurodegenerative diseases [140, 141]. More than 160 rare diseases have been identified, most of which are inherited in an autosomal recessive manner. These disorders are caused by defects in glycosylation patterns and are collectively known as congenital disorders of glycosylation (CDG) [142].

Dol-P is also involved in glycosylphosphatidylinositol (GPI) biosynthesis, where it serves as a membrane-bound scaffold in the endoplasmic reticulum for the stepwise assembly of the GPI precursor. The core glycan is constructed on Dol-P through the sequential addition of sugar residues and other components on the luminal side of the ER membrane. Once fully assembled, the preformed GPI anchor is transferred en bloc to specific proteins by a GPI transamidase complex, resulting in their covalent attachment to the outer leaflet of the plasma membrane [143].

Pren-P is essential in bacterial glycan biosynthesis, particularly in the formation of the peptidoglycan cell wall. In bacteria, undecaprenyl phosphate functions as a lipid carrier for peptidoglycan precursors, including Lipid I and Lipid II [144]. These intermediates are assembled in the cytoplasm and subsequently translocated across the cytoplasmic membrane via a lipid-linked mechanism. Following their incorporation into the growing peptidoglycan network on the extracellular side, the carrier lipid is dephosphorylated and recycled, enabling continuous cell wall synthesis [93].

### Protein Prenylation

While Dol-P and Pren-P function as lipid carriers for glycans in protein glycosylation, short-chain isoprenoid lipids, FPP and GGPP, serve as donors of isoprenoid groups during protein prenylation in various species. This post-translational modification involves the covalent attachment of hydrophobic farnesyl or geranylgeranyl groups to cysteine residues located near the C-terminus of target proteins, which promotes membrane association and modulates their regulatory activity [145].

Prenylation is mediated by three specific enzymes: farnesyltransferase (FT), geranylgeranyltransferase type I (GGT I), and Rab geranylgeranyltransferase (RGGT, also known as GGT II) [146, 147]. Most substrates are recognized via a C-terminal CaaX motif, where C is the modified cysteine, a1 and a2 are aliphatic residues, and the identity of the X residue determines whether the protein is farnesylated or geranylgeranylated [145, 148]. If X is serine, methionine, alanine, or glutamine, the protein is farnesylated, whereas leucine or isoleucine typically directs geranylgeranylation [145]. Proteins such as members of the Ras superfamily (H-Ras, N-Ras, and K-Ras) and nuclear lamins are well-documented substrates for farnesylation [147, 149], while geranylgeranylation primarily modifies small GTPases of the Rho and Rab families [149]. Notably, some proteins, such as RhoB, can undergo both modifications depending on cellular context [150, 151].

These modifications are essential for the control of cellular processes that maintain homeostasis, support development, and enable responses to environmental signals. It increases protein hydrophobicity, facilitating membrane association and proper biological activity of diverse regulatory proteins, including GTPases, G proteins, and nuclear lamins. Prenylation is therefore critical for key cellular processes such as signaling, growth, development, and survival across species. In humans, disturbances in prenylation are associated with cardiovascular, neurological, and neurodegenerative diseases [152, 153], while in plants prenylated proteins play important roles in adaptation to environmental stresses such as drought, salinity, high temperature, and heavy metal exposure, as well as in defense against pathogens [154, 155].

### Protein Dolichylation

While protein prenylation involves the attachment of short-chain isoprenoid groups to target proteins, long-chain isoprenoid lipids can also participate in protein modification via dolichylation. It is a unique and still poorly understood post-translational modification characterized by the covalent attachment of long-chain dolichols to cellular proteins [156, 157]. This mechanism was initially identified in three human tumor cell lines, including colon carcinoma, melanoma, and hepatoblastoma cells [156]. Subsequent studies have also demonstrated its occurrence in the human malaria parasite *Plasmodium falciparum* [157]. Although the precise biological function of protein dolichylation remains unclear, available evidence suggests a potential role in the regulation of the cell cycle, intracellular protein targeting, and membrane anchoring processes [157]. Despite these findings, many aspects of protein dolichylation remain unresolved. The enzymes responsible for the transfer of dolichols to protein substrates have not been identified yet. In addition, the identity of the activated lipid donor, as well as target proteins, their subcellular localization and biological roles, remain largely unknown, leaving open space for further investigation.

### Quinone Prenylation

In addition to proteins, quinones represent another class of prenylated compounds. Prenylquinones are characterized by a quinone core substituted with a hydrophobic isoprenoid side chain and are ubiquitous in almost all living organisms [158]. The biosynthesis of prenylquinones involves the condensation of a Pren-PP chain with an aromatic quinone ring [158, 159]. This process is essential, as the hydrophobic isoprenoid tail determines lipid solubility and anchors the quinone core within the membrane bilayer [158]. This stable membrane association enables prenylquinones to function as mobile redox carriers, facilitating electron and proton transfer between enzymatic complexes and maintaining the electrochemical gradients required for ATP synthesis [160–162]. The major classes of biological prenylquinones include ubiquinone (UQ), menaquinone (MK), plastoquinone (PQ), and rhodoquinone (RQ). Ubiquinones typically range from UQ-8 in bacteria to UQ-10 in yeast and humans, whereas menaquinones exhibit the widest variability (MK-4 to MK-13 across bacteria, archaea, and mammals). Plastoquinone is predominantly represented by PQ-9 in plants and algae, while rhodoquinone most commonly occurs as RQ-9 in bacteria and anaerobic invertebrates [158]. The length of the isoprenoid side chain varies significantly across species and is adapted to specific membrane environments and functional requirements. Organisms utilize different chain lengths to optimize interactions with binding partners and to match the lipid composition of their membranes. Side chain length directly influences membrane localization: long-chain ubiquinones are embedded within the hydrophobic core of membranes, where they support efficient electron and proton transfer across the bilayer. In contrast, short-chain quinones tend to localize closer to the polar membrane-water interface [163]. Functionally, these molecules act as essential mobile carriers in respiratory and photosynthetic electron transport chains [159, 162, 164]. Beyond bioenergetics, they also function as important antioxidants, protecting membranes from lipid peroxidation [165]. The final chain length is determined by CPTs through specialized product-binding pockets and structural length-determining mechanisms [158, 166]. These enzymes employ conserved motifs that control and terminate elongation at a defined number of isoprene units. Despite substantial progress in understanding prenylquinone structure and function, the regulation of species-specific side-chain length and its adaptation to different physiological or environmental conditions is still not fully resolved.

### Effect on Lipid Membrane Properties

Beyond their established roles as lipid carriers in protein glycosylation and as donors of isoprenoid groups in protein and quinone prenylation, polyisoprenoids act as important modulators of biological membrane properties, influencing membrane fluidity, permeability, and overall membrane dynamics [26].

Biophysical studies using model membrane systems have demonstrated that polyisoprenoids significantly affect lipid bilayer organization and phospholipid packing [11]. Their incorporation is associated with increased membrane fluidity and permeability, as well as large-scale reorganizations of membrane structure. A key feature of these effects is the promotion of non-lamellar lipid organization, in particular the inverted hexagonal phase (HII), which is characterized by high curvature and reduced bilayer stability and is generally linked to enhanced membrane dynamics, including fluidity, permeability, and fusogenicity [11, 167, 168]. The effects of polyisoprenoids are strongly dependent on membrane lipid composition. In phosphatidylethanolamine (PE)-rich systems, dolichols promote destabilization of the bilayer structure and facilitate the formation of non-lamellar phases. In contrast, in phosphatidylcholine (PC) membranes, their effects are weaker and they tend to partition into membrane domains without strongly disturbing the overall bilayer organization. Phosphorylated derivatives such as Dol-P can exert more pronounced effects on membrane structure and lipid dynamics than free dolichols [11]. In addition, polyprenols have been shown to increase membrane conductance and permeability, further indicating their role in the regulation of membrane transport properties [169].

A related example of polyisoprenoid behavior in membranes is provided by archaeal systems, where the non-polar polyisoprenoid squalene localizes in the bilayer midplane, oriented perpendicular to membrane lipids. This positioning increases membrane thickness and fluidity while simultaneously acting as a barrier to proton leakage. Maintenance of low proton permeability is critical for sustaining proton gradients and ATP production under extreme temperature or hydrostatic pressure conditions [170].

Importantly, the functional effects of polyisoprenoids also depend on their molecular structure. Ciepichal et al. [168] showed that *cis/trans* isomerization of the α-residue of polyprenols significantly influences both membrane permeability and thermotropic behavior in model phospholipid–polyisoprenoid systems. In phosphatidylcholine bilayers, α-*trans* polyprenols increased glucose permeability by up to 1.7-fold, whereas α-*cis* isomers had little or no effect. These findings demonstrate that relatively small structural differences within polyisoprenoid molecules can lead to pronounced changes in their interaction with lipid bilayers and in their effects on membrane properties.

The ability of polyisoprenoids to modulate membrane organization has important physiological consequences. In plant thylakoid membranes, polyprenols contribute to the maintenance of proper membrane structure and support efficient photosynthetic activity, whereas their deficiency leads to membrane disorganization, impaired photosystem II function, and reduced photosynthetic electron transport [35].

Overall, polyisoprenoids act as dynamic regulators of membrane architecture, with their effects determined by both membrane lipid composition and their own molecular structure. Through their ability to modulate lipid packing, promote non-lamellar phases, and alter permeability, they play an important role in controlling the physical properties of biological membranes essential for cellular function.

Beyond their role in shaping lipid organization, increasing evidence suggests that polyisoprenoids may also participate in the spatial organization of membrane-associated proteins. Specific membrane proteins contain polyisoprenyl recognition sequences (PIRS) that mediate selective interactions with polyisoprenoids. Through these interactions, polyisoprenoids may act as structural scaffolds that help organize and anchor proteins within specialized membrane domains [171, 172]. Interestingly, such interactions may also influence membrane architecture, as binding of polyisoprenoids to PIRS-containing proteins has been proposed to reverse non-lamellar structures and stabilize lamellar bilayers [171].

### Protection Against Pathogens and Stress Responses

While polyisoprenoids are key modulators of membrane properties such as fluidity and organization, these biophysical effects are closely linked to their role in cellular stress adaptation and defense. By influencing membrane stability and dynamics, polyisoprenoids contribute to the maintenance of cellular homeostasis and enhance organismal resilience to environmental challenges, including high temperature, salinity, osmotic stress, and pathogen attack. Beyond their structural roles, both polyprenols and dolichols are involved in physiological defense systems, contributing to protection against pathogens and stress responses. They have been proposed to act as antioxidants and free-radical scavengers, capable of neutralizing reactive oxygen species and limiting lipid peroxidation [62]. Their levels change in response to changing environmental conditions. For example, light plays a major role in regulating isoprenoid metabolism in plants by enhancing the plastidial MEP pathway, leading to polyprenol accumulation [173], while high temperature promotes their accumulation in thylakoid membranes to stabilize membrane architecture and protect photosynthetic protein complexes from thermal degradation [128]. Increased levels are also observed under salinity and osmotic stress [174]. Biotic stress, particularly pathogen infection, also triggers a defensive accumulation of polyisoprenoids. In *N. tabacum*, infection by Tobacco Mosaic Virus (TMV) or *Pseudomonas syringae* leads to a measurable increase in polyprenols and solanesol levels [130]. This protective capacity is important for plants for surviving environmental pressures such as drought, extreme temperatures, and endoplasmic reticulum stress. Overall, polyisoprenoids are integral to membrane function, protein modification, and cellular defense. Their accumulation is closely associated with stress responses and aging. Despite their importance, the precise molecular mechanisms underlying their roles in stress tolerance and cellular regulation remain not fully understood and are still under active investigation.

## Disruption of the Dolichol Biosynthetic Pathway and its Consequences in Humans

Mutations affecting genes involved in dolichol biosynthesis in humans lead to congenital disorders of glycosylation (CDG), a heterogeneous group of inherited metabolic diseases caused by defects in protein and lipid glycosylation [115, 133]. These disorders are typically multisystemic and arise from impaired processing of complex carbohydrates. Historically, CDG were classified into type I and type II based on the subcellular localization of the defect. CDG type I result from impaired synthesis of the dolichol-linked oligosaccharide precursor or its transfer to nascent proteins in the endoplasmic reticulum, whereas CDG type II are associated with defective processing of protein-bound glycans in the Golgi apparatus [115]. More recently, CDG have been reclassified into broader categories that include defects in N-linked glycosylation, O-linked glycosylation, combined glycosylation pathways, and glycosylphosphatidylinositol (GPI) anchor biosynthesis [175].

Genetic defects affecting individual steps of dolichol biosynthesis give rise to clinically distinct disorders with variable severity and tissue involvement. Mutations in *NUS1* cause NUS1-CDG, characterized by congenital scoliosis, refractory epilepsy, and severe neurological impairment [21]. In contrast, DHDDS-associated disease was initially linked to isolated retinitis pigmentosa but is now recognized as a broader multisystem disorder that may include hypotonia, hepatomegaly, and sensorineural deafness [176]. Importantly, biallelic DHDDS K42E variants associated with autosomal recessive retinitis pigmentosa (RP59) demonstrate that pathological consequences of this pathway may result not from dolichol deficiency itself, but from alterations in dolichol chain-length composition. Patients carrying the K42E/K42E substitution exhibit a shift from the predominant Dol-19 species toward shorter dolichols, such as Dol-17 and Dol-18, despite the absence of abnormalities in serum transferrin glycosylation, indicating that altered dolichol composition alone may contribute to retinal degeneration [176–178].

Additional disorders affect later stages of the pathway, including the conversion of polyprenol to dolichol. DHRSX-CDG and SRD5A3-CDG disrupt this process and lead to accumulation of polyprenol intermediates, such as polyprenal and polyprenol, which impair downstream N-glycosylation [23, 179]. DHRSX-CDG is associated with severe intellectual disability, epilepsy, and characteristic facial dysmorphism, whereas SRD5A3-CDG typically presents with ocular abnormalities, brain malformations, and dermatological manifestations [23]. Not only dolichol deficiency, but also excessive polyprenol accumulation, can lead to disease pathology. In an SRD5A3-CDG patient, dolichol levels remain relatively preserved, while unreduced polyprenols can accumulate approximately threefold, resulting in a markedly increased polyprenol/dolichol ratio. This suggests that disruption of polyisoprenoid homeostasis may be pathogenic even in the absence of dolichol depletion. Importantly, excess polyprenol is thought to impair N-glycosylation by competing with dolichol during the assembly of dolichol-linked oligosaccharides [179].

Deficiency of DOLK, the enzyme responsible for phosphorylation of dolichol to Dol-P, causes DOLK-congenital disorders of glycosylation (DOLK-CDG), a severe form of CDG type I characterized by impaired protein glycosylation [123, 180]. Biallelic mutations in the *DOLK* gene markedly reduce Dol-P availability, thereby disrupting glycoprotein biosynthesis and leading to severe multisystem disease. Clinically, DOLK-CDG is most commonly associated with progressive dilated cardiomyopathy, severe ichthyosis, hypotonia, seizures, liver dysfunction, and neurological impairment, frequently resulting in early childhood mortality [180–183]. The severity of the DOLK-CDG phenotype highlights the essential role of dolichol phosphorylation in cellular homeostasis, as profound defects in glycosylation compromise fundamental cellular processes required for normal organismal function. At the cellular level, impaired dolichol metabolism promotes accumulation of misfolded glycoproteins in the endoplasmic reticulum, triggering the unfolded protein response (UPR) and inflammatory signaling pathways. Furthermore, altered dolichol homeostasis has been implicated in aging-related cellular dysfunction and has been proposed as a contributing factor in neurodegenerative diseases, including Alzheimer’s disease [184].

## Polyisoprenoids in Diagnostics and Disease Treatments

The severe clinical consequences of disrupted polyisoprenoid metabolism have highlighted their involvement in a broad spectrum of genetic disorders and stimulated interest in their potential diagnostic and therapeutic applications. Polyisoprenoids have therefore emerged as biomolecules of clinical relevance, both as potential biomarkers and as candidates for therapeutic use in various pathological conditions. Ropren is a plant-derived polyprenol formulation composed of a mixture of long-chain polyprenols isolated from the needles of *Pinus sibirica* and related Pinus species, which has shown clinical benefits in reducing anxiety, depression, and cognitive impairment in patients with Alzheimer’s disease and acute coronary syndrome [185, 186]. In addition, polyprenol-based preparations have been used as hepatoprotective agents in liver disorders such as fatty liver disease, hepatitis, and cirrhosis, as well as in mitigating drug- or alcohol-induced liver injury [185, 187]. In cardiovascular disease, Ropren has been reported to reduce total cholesterol levels and to protect against liver damage associated with high-dose lipid-lowering therapies [187]. Supplementation with high doses of polyprenols has also been associated with improvement of skin conditions, particularly atopic dermatitis [188]. Dolichols and their phosphate derivatives have been investigated in the context of metabolic disorders, including effects on glucose homeostasis in diabetes and uric acid levels in gout. They have also been reported to support hepatic function and regeneration following liver injury or surgery [189].

Dolichol profiles serve as informative biomarkers for genetic disorders such as retinitis pigmentosa, where a characteristic shift from Dol-19 to Dol-18 reflects impaired biosynthesis associated with *DHDDS* mutations [178]. More broadly, dolichols are considered as biomarkers of mammalian aging due to their progressive accumulation in tissues over time [52, 57]. Recent studies have further refined this view, with analyses of mouse retina revealing a pronounced biphasic increase in dolichol species, with levels rising up to 100-fold from birth to late adulthood [52]. Another study introduces “DoliClock,” a lipid-based aging model showing that prefrontal cortex dolichol levels can accurately predict biological age [190]. This approach has also revealed signs of accelerated biological aging in neurological disorders, including autism, schizophrenia, and Down syndrome. This accumulation of dolichols is thought to reflect their high chemical stability and slow turnover rather than increased synthesis [52, 190]. However, key questions remain regarding the functional consequences of this accumulation, including whether it contributes to membrane protection against oxidative stress or UV-induced damage [52]. Further studies addressing the metabolic regulation of dolichol dolichol homeostasis may provide new insights into their role in aging and age-related diseases.

## Future Directions

Future polyisoprenoid studies should increasingly move beyond descriptive chemical and biological characterization toward predictive and integrated metabolic engineering approaches. Advanced structural biology techniques, including cryo-electron tomography (cryo-ET), now enable visualization of protein complexes within their native membrane environments [191–193]. In parallel, AI-based tools such as AlphaFold are increasingly being used to predict the organization of large enzyme assemblies [191, 194, 195]. To further investigate protein dynamics, generative diffusion models such as BioEmu can predict conformational landscapes and their responses to ligand binding [194]. Together, these approaches may significantly improve our understanding of heteromeric CPT complexes and the mechanisms governing polyisoprenoid biosynthesis. Recent advances in synthetic biology have also demonstrated the feasibility of large-scale production of complex terpenoids, including diterpenoids such as taxol, a widely used anticancer drug, using engineered microbial systems [196]. These developments highlight the broader potential of heterologous platforms for sustainable and scalable production of valuable isoprenoid-derived molecules, including polyisoprenoids. Another important direction involves plant engineering aimed at improving resistance to changing environmental conditions. CRISPR/Cas-mediated prime editing and promoter engineering now allow precise and transgene-free metabolic modulation in crops [197]. Because polyisoprenoids influence membrane fluidity and stabilize thylakoid architecture under stress conditions, modulation of CPT activity may enhance the ability of plants to maintain membrane homeostasis during environmental fluctuations. For example, increasing specific polyisoprenoid levels could stabilize thylakoid membranes and protect photosynthetic machinery during heat stress.

Machine learning platforms are also being applied to predict the pharmacological activities and toxicity profiles of polyisoprenoid derivatives. In addition, high-throughput screening approaches based on microfluidic “mix-and-spray” systems enable rapid identification of ligand-induced conformational changes in biomolecular complexes [192]. Collectively, these emerging technologies provide a foundation for deeper understanding of polyisoprenoid pathways and may facilitate the development of novel compounds and engineering strategies with improved therapeutic and agricultural potential.

## Discussion and Conclusions

Despite considerable advances in the field of polyisoprenoid research, several key questions regarding their metabolism, regulation, and cellular functions remain unresolved. These challenges span multiple levels of biological organization, from precursor biosynthesis and enzyme regulation to pathway integration and physiological function.

A primary area of current interest is the metabolic crosstalk between MVA and MEP pathways, which provide the five-carbon precursors for all polyisoprenoids. While “mosaic isoprenoids” demonstrate that these pathways are functionally integrated in plants, many aspects of their interaction remain unresolved. For instance, the mechanisms governing the transport of intermediates such as IPP across the plastidial membrane are still unclear, as no specific transporters have been identified to date. Furthermore, although this metabolic interplay provides flexibility and enables carbon redistribution in response to light or developmental cues, it is not sufficient to fully compensate for the loss of either pathway, as mutations in both systems are typically lethal.

Another important challenge is the regulation of CPT activities. Current structural models propose a “molecular ruler” mechanism, where the size of a hydrophobic cleft determines product chain length [82]. However, these models do not fully account for how external factors, such as metal ion availability or membrane lipid composition, influence enzymes selectivity. While heteromeric CPT complexes require accessory subunits (CPT-BP) for activity, the molecular basis of their regulation and catalytic timing remains incompletely understood. This is particularly evident in natural rubber biosynthesis, where CPTs function as part of large multimeric assemblies responsible for the production of extremely long polyisoprenoid chains [111]. How similar high-order assemblies operate in other biological systems remains unresolved.

Beyond structural determinants, the post-transcriptional regulation of CPTs represents another significant knowledge gap. Although these enzymes are essential for cell viability, the mechanisms controlling their stability, activity, and integration into lipid metabolic networks remain poorly characterized. Consequently, understanding how CPTs contribute to lipid homeostasis during development and environmental stress remains an important research direction.

A recent paradigm shift concerns the conversion of polyprenols into dolichols. For decades, this was considered a single-step reduction process. However, recent evidence has proposed a three-step pathway involving aldehyde intermediates. In humans, DHRSX has been suggested to catalyze both oxidation of polyprenol to polyprenal and reduction of dolichal to dolichol [23]. However, the situation in yeast remains debated. While some studies propose that Tda5 serves as the sole ortholog performing both functions, others suggest a two-enzyme system involving Tda5 and Env9 [24, 25]. Resolving this discrepancy is essential for a complete understanding of dolichol metabolism.

Over the past 70 years, polyisoprenoid research has evolved from the initial discovery of solanesol in 1956 to the characterization of complex heteromeric enzyme systems and multi-step biosynthetic pathways. These compounds are now recognized not as secondary metabolites, but as essential lipid carriers in glycosylation, structural components of membranes, and key contributors to stress responses. Despite these advances, important mechanistic questions remain unanswered, and addressing them will be essential for a comprehensive understanding of polyisoprenoid biology.

## Data Availability

No datasets were generated or analysed during the current study.
